# Tocilizumab Efficacy Across Inflammatory Subphenotypes in COVID-19-Related Acute Respiratory Distress Syndrome

**DOI:** 10.1097/CCE.0000000000001392

**Published:** 2026-03-16

**Authors:** Daan F. L. Filippini, Jessica Khyali, Malou Janssen, Emma Rademaker, Olaf L. Cremer, Tom van der Poll, Rombout B. E. van Amstel, Henrik Endeman, Lieuwe D. J. Bos, on behalf of the Amsterdam UMC COVID-19 Biobank Study Group, Anne Geke Algera

**Affiliations:** Department of Intensive Care; Department of Infectious Diseases; Department of Intensive Care; Department of Neurology; Department of Neurology; Department of Pulmonology; Department of Intensive Care; Department of Intensive Care; Department of Infectious Diseases; Department of Infectious Diseases; Department of Neurology; Department of Intensive Care; Department of Pathology; Department of Intensive Care; Department of Infectious Diseases; Experimental Immunology; Department of Infectious Diseases; Department of Clinical Chemistry; Department of Clinical Chemistry; Department of Intensive Care; Department of Intensive Care; Department of Intensive Care; Department of Intensive Care; Department of Infectious Diseases; Department of Experimental Immunology; Department of Intensive Care; Department of Infectious Diseases; Department of Infectious Diseases; Department of Intensive Care; Amsterdam UMC Biobank Core Facility; Department of Infectious Diseases; Department of Radiology; Department of Infectious Diseases; Department of Intensive Care; Department of Anesthesiology; Department of Intensive Care; Department of Infectious Diseases; Department of Medical Microbiology; Department of Neurology; Department of Intensive Care; Department of Intensive Care; Department of Infectious Diseases; Department of Pulmonology; Department of Intensive Care; Department of Infectious Diseases; Department of Neurology; Department of Infectious Diseases; Department of Anesthesiology; Department of Intensive Care; Department of Infectious Diseases; Department of Infectious Diseases; Department of Clinical Chemistry; Department of Intensive Care; Department of Internal Medicine; Department of Intensive Care; Department of Infectious Diseases; Department of Intensive Care and Infectious Diseases; Department of Pulmonology; Department of Intensive Care; Department of Infectious Diseases; Department of Intensive Care; Neurochemical Laboratory; Department of Intensive Care; Department of Intensive Care; Department of Intensive Care; Department of Infectious Diseases; Department of Anesthesiology; Department of Intensive Care; Department of Intensive Care; Department of Intensive Care; Department of Intensive Care and Infectious Diseases; Department of Infectious Diseases; Department of Infectious Diseases; Department of Clinical Chemistry; Department of Clinical Epidemiology, Biostatistics and Bioinformatics; 1 Department of Intensive Care Medicine, Amsterdam UMC, University of Amsterdam, Amsterdam, The Netherlands.; 2 Department of Intensive Care Medicine, Erasmus MC, Erasmus University Rotterdam, Rotterdam, The Netherlands.; 3 Department of Internal Medicine, Erasmus MC, Erasmus University Rotterdam, Rotterdam, The Netherlands.; 4 Department of Intensive Care Medicine, UMC Utrecht, Utrecht University, Utrecht, The Netherlands.; 5 Julius Center for Health Sciences and Primary Care, UMC Utrecht, Utrecht, The Netherlands.; 6 Center for Infection and Molecular Medicine (C.I.M.M.), Amsterdam UMC, University of Amsterdam, Amsterdam, The Netherlands.; 7 Division of Infectious Diseases, Amsterdam UMC, University of Amsterdam, Amsterdam, The Netherlands.; 8 Department of Intensive Care Medicine, OLVG, Amsterdam, The Netherlands.; 9 Department of Pulmonology, Amsterdam UMC, University of Amsterdam, Amsterdam, The Netherlands.; 10 Laboratory of Experimental Intensive Care and Anesthesiology (L.E.I.C.A.), University of Amsterdam, Amsterdam, The Netherlands.

**Keywords:** COVID-19, inflammatory subphenotypes, mechanical ventilation, mortality, tocilizumab

## Abstract

**OBJECTIVES:**

This study evaluated whether the established efficacy of tocilizumab, an interleukin-6 (IL-6) receptor antagonist, differs between the hypoinflammatory and hyperinflammatory subphenotypes.

**DESIGN:**

Retrospective analysis of data from three biobanks.

**SETTING:**

ICUs of three university teaching hospitals in the Netherlands.

**PATIENTS:**

Mechanically ventilated patients with COVID-19.

**INTERVENTIONS:**

Tocilizumab administration vs. no administration.

**MEASUREMENTS AND MAIN RESULTS:**

A total of 561 patients were included. Based on a classifier model incorporating IL-6, tumor necrosis factor receptor 1, and bicarbonate, 95% were classified as Hypoinflammatory and 5% as Hyperinflammatory. Tocilizumab was associated with a significant reduction in 30-day mortality in the overall cohort, even after adjustment for confounders (*p* = 0.014). However, there was no evidence that treatment effectiveness differed between the two subphenotypes (*p* = 0.59).

**CONCLUSIONS:**

In this cohort, tocilizumab significantly reduced 30-day mortality overall. Although the number of Hyperinflammatory patients was low, there was no evidence that its efficacy differed between inflammatory subphenotypes. These findings underscore the importance of including both subphenotypes in future trials evaluating the differential effects of tocilizumab.

**KEY POINTS**

**Question**: This study aimed to determine whether the established efficacy of tocilizumab, an interleukin-6 receptor antagonist, differs between hypoinflammatory and hyperinflammatory subphenotypes in mechanically ventilated COVID-19 patients.

**Findings**: In a retrospective analysis of 561 patients from three university hospital ICUs, tocilizumab administration was associated with a significant reduction in 30-day mortality in the overall cohort. Although only 5% of patients was classified as hyperinflammatory and 95% as hypoinflammatory, we found was no evidence that tocilizumab’s effect differed between the two inflammatory subphenotypes.

**Meaning**: These results emphasize the value of including both subphenotypes in future trials assessing targeted immunomodulatory therapies.

Tocilizumab, an interleukin (IL)-6 inhibiting monoclonal antibody, reduces mortality in patients with severe COVID-19 (1). The baseline immune profile of COVID-19 patients predicts clinical outcomes but it remains unclear if the immune profile moderates the effectiveness of tocilizumab. In patients with acute respiratory distress syndrome (ARDS) due to other causes than COVID-19, inflammatory subphenotypes largely based on profiles of pro-inflammatory plasma biomarkers, have been identified (2). Patients with a hyperinflammatory subphenotype consistently have worse outcomes and differential treatment responses were identified for a wide range of interventions (3). Resolution of this hyperinflammatory subphenotype is an important pathway toward recovery in critically ill patients with ARDS and sepsis (4). Multiple parsimonious classifier have demonstrated accurate and rapid identification of inflammatory subphenotypes, one of which is based on plasma concentrations of IL-6, tumor necrosis factor receptor 1 (TNFR1), and bicarbonate (5). Using this prior knowledge and pathophysiological reasoning, we hypothesized that the effect of tocilizumab on mortality differs between inflammatory subphenotypes in patients with COVID-19-related ARDS, with a beneficial effect in the hyperinflammatory subphenotype and little or no effect in the hypoinflammatory subphenotype.

## METHODS

We analyzed data from three biobanks of academic hospitals in the Netherlands: Amsterdam UMC (center 1), UMC Utrecht (center 2), and Erasmus MC (center 3). Detailed information on Institutional Review Board approval is provided in **Appendix A**. These biobanks contain plasma samples from patients who were mechanically ventilated primarily due to COVID-19. On the day of ICU admission, plasma samples were collected and later batch-analyzed to derive IL-6 and TNFR1 values, using Luminex (Luminex Corporation, Austin, TX) in centers 1 and 2 and the ELLA platform (Simple Plex; Bio-Techne, Minneapolis, MN) at center 3. Interassay variability was assessed, and no significant differences were observed. An existing biomarker classifier based on IL-6, TNFR1, and bicarbonate levels was used to assign inflammatory subphenotype probabilities, ranging from 0 (highest probability hypoinflammatory) to 1 (highest probability hyperinflammatory) (5). A cutoff of 0.5 was used for dichotomous classification, and continuous probabilities were also analyzed, making the exact threshold less critical.

In the Netherlands, tocilizumab was administered in combination with corticosteroids, following national guidelines. These guidelines selected patients with moderate to severe disease, defined by increasing oxygen demand and elevated C-reactive protein (CRP) levels. Consequently, treatment was usually initiated shortly before ICU admission. To account for baseline patient characteristics on which tocilizumab administration may have been based, patients treated with tocilizumab were matched 1:2 to untreated controls based on age, sex, CRP levels, and Pao_2_/Fio_2_, using nearest-neighbor propensity score matching. Missing data (< 1%) in matching variables were imputed using multivariable imputation by chained equations, assuming data were missing at random. The primary outcome was the interaction between inflammatory subphenotype probability and tocilizumab treatment, with 30-day mortality as the dependent variable. A logistic regression model was used to predict 30-day mortality, including subphenotype probability, treatment allocation, their interaction term and covariates corticosteroid use, inclusion center and COVID wave to adjust for potential confounding. The *p* value for the interaction term was derived from the Wald test.

## RESULTS

### Complete Cohort

A total of 561 patients were included, with 30 (5%) classified as hyperinflammatory and 531 (95%) as hypoinflammatory. Tocilizumab was administered more frequently to hyperinflammatory patients than to hypoinflammatory patients (53% vs. 29%; *p* = 0.008), despite similar CRP levels (116 [interquartile range (IQR), 28–219] vs. 105 [IQR, 36–209]; *p* = 0.64). Furthermore, hyperinflammatory patients had a significantly higher 30-day mortality (odds ratio [OR], 2.78; 95% CI, 1.32–5.86; *p* = 0.007).

### Matched Cohort

**Figure 1.**
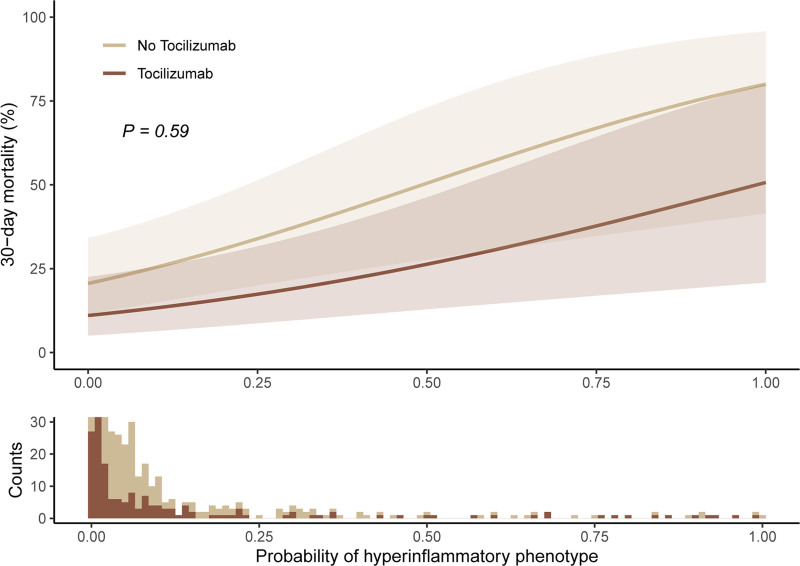
Predicted 30-d mortality by treatment group and probability of hyperinflammatory subphenotype. A logistic regression model was used to predict 30-d mortality, with the probability of belonging to the hyperinflammatory subphenotype (*x*-axis), treatment group (tocilizumab vs. matched control), their interaction term and confounders (corticosteroid administration, inclusion center, and COVID wave) as predictor variables. The *lines* represent the estimated probability of 30-d mortality for each treatment group, with *shaded areas* indicating the 95% CIs. The *p* value reflects the Wald test for the interaction between subphenotype probability and tocilizumab treatment in the model. The **lower** displays histograms of patient counts stratified by treatment group across the range of hyperinflammatory subphenotype probabilities, illustrating the distribution of patients in both the tocilizumab and control groups.

Tocilizumab was administered to 172 patients (31%), who were matched to 344 untreated controls (**Table 1**). Covariate balance was achieved after matching, with standardized mean differences below 0.1 for all matching variables (sex, CRP, Pao_2_/Fio_2_) except age (0.12). Tocilizumab-treated patients had a significantly lower 30-day mortality than matched controls, even after adjusting for corticosteroid treatment, inclusion center, and COVID wave (OR, 0.57; 95% CI, 0.37–0.94; *p* = 0.014). This held up in a subgroup of hypoinflammatory patients (*p* = 0.014) but not in hyperinflammatory patients (*p* = 0.15). No significant interaction was observed between inflammatory subphenotype probability and tocilizumab treatment effect (estimate, –0.72; 95% CI, –2.90 to 1.46; *p* = 0.51) which remained after adjusting for corticosteroids, inclusion center, and COVID wave (estimate, –0.62; 95% CI, –2.89 to 1.64; *p* = 0.59; **Fig. 1**), indicating that the efficacy of tocilizumab does not appear to depend on baseline inflammatory subphenotype. A sensitivity analysis excluding patients not receiving corticosteroids yielded similar results (estimate, –0.72; 95% CI, –2.30 to 1.21; *p* = 0.63), and an analysis using only baseline IL-6 (*p* = 0.23) or baseline CRP (*p* = 0.68) levels instead of probability scores also showed no significant findings.

**TABLE 1.** Baseline Characteristics of Tocilizumab and Matched Controls

Variable	Tocilizumab (*n* = 172)	Matched Controls (*n* = 344)	Standardized Mean Difference	*p*
Demographics				
Age	62 (52–68)	62 (54–70)	0.12	0.19
Male (%)	118 (69%)	227 (66%)	0.06	0.62
Body mass index	29.4 (25.8–34.0)	29.1 (25.7–32.9)	0.08	0.59
Center			0.28	0.010
1 (%)	57 (33%)	74 (22%)		
2 (%)	40 (23%)	79 (22%)		
3 (%)	75 (44%)	191 (56%)		
Wave and strain^a^			1.10	< 0.001
1—Original (%)	1 (1%)	160 (31%)		
2—Original (%)	37 (22%)	95 (28%)		
3—Alpha (%)	110 (64%)	87 (27%)		
4—Delta (%)	24 (14%)	52 (15%)		
Comorbidities				
Chronic pulmonary disease (%)	16 (9%)	25 (7%)	0.07	0.53
Diabetes (%)	48 (28%)	88 (26%)	0.05	0.65
Hypertension (%)	35 (36%)	70 (46%)	0.20	0.19
Immune compromised (%)	22 (13%)	41 (12%)	0.03	0.89
Severity				
Acute Physiology and Chronic Health Evaluation IV	55 (42–65)	58 (52–70)	0.35	0.13
Sequential Organ Failure Assessment score	6 (4–7)	6 (5–8)	0.37	0.001
Lactate (mmol/L)	1.40 (1.17–1.65)	1.50 (1.20–2.05)	0.37	0.098
C-reactive protein (mg/L)	92 (26–157)	87 (30–173)	0.05	0.51
Pao_2_/Fio_2_ (mm Hg)	146 (98–204)	150 (113–204)	0.04	0.30
Medication				
Corticosteroids (%)	172 (100%)	250 (74%)	0.84	< 0.001
Subphenotype				
Interleukin-6 (pg/mL)	173 (67–584)	74 (25–320)	0.15	< 0.001
Tumor necrosis factor receptor 1 (pg/mL)	2339 (1848–3298)	2682 (1959–3899)	0.19	0.014
Bicarbonate (mmol/L)	25.1 (22.4–27.7)	25.45 (22.8–29.0)	0.15	0.193
Hypoinflammatory (%)	153 (90%)	334 (97%)	0.21	0.005
Probability^b^	0.03 (0.01–0.12)	0.02 (0.00–0.07)	0.31	< 0.001

a The most dominant COVID-19 strain in the Netherlands.

b Subphenotype probability assigned using a well-validated classifier previously published, based on interleukin-6, tumor necrosis factor receptor 1, and bicarbonate.

Values are presented as median (interquartile range), unless indicated as (%), in which case they are shown as *n* (%).

## DISCUSSION

In our cohort, which consisted predominantly of hypoinflammatory COVID-ARDS patients, tocilizumab was associated with a reduction in 30-day mortality overall, but we found no evidence of a differential effect between the hypoinflammatory and hyperinflammatory subphenotypes. While prior studies have suggested that hyperinflammatory states may predict greater responsiveness to immunomodulatory therapies (6, 7), our findings indicate that tocilizumab may be effective even in primarily hypoinflammatory populations.

The low prevalence of the hyperinflammatory subphenotype in our cohort aligns with findings from other COVID-19 ARDS studies, indicating that severe COVID-19 is predominantly associated with a hypoinflammatory phenotype (8). By using continuous probability scores in our main analysis, we preserved more information and reduced the dependence on the specific cutoff or classifier model used. We also confirmed the prognostic value of the hyperinflammatory subphenotype, and the double risk for death is consistent with previous studies in ARDS and sepsis (2, 9). Notably, hyperinflammatory patients were more likely to receive tocilizumab despite having similar CRP levels compared with hypoinflammatory patients, suggesting that physicians somehow preselected patients with hyperinflammatory features.

Some limitations should be acknowledged. First, the small number of hyperinflammatory patients markedly limited our statistical power to detect subtle interaction effects. Second, residual confounding may persist despite adequate matching, particularly due to unknown vaccination statuses or temporal factors, such as variations in COVID-19 strains and the absence of tocilizumab as a treatment during the first waves. Such confounding could bias our results by artificially inflating the apparent benefit of tocilizumab, as treated patients were predominantly from later waves when outcomes generally improved due to evolving clinical practices. Third, biomarker measurements were confined to admission samples, potentially overlooking dynamic changes in inflammatory status influenced by tocilizumab over time (4, 10). Additionally, as an IL-6R antibody, tocilizumab may alter IL-6 levels, although this effect typically emerges after several days (11).

In conclusion, while tocilizumab’s effect on mortality may not differ by inflammatory subphenotype in critically ill COVID-19 patients, this should be interpreted cautiously given the small number of hyperinflammatory patients. Importantly, in the larger hypoinflammatory group, where statistical power is greater, a beneficial effect of tocilizumab on mortality is still observed. This finding has implications for future randomized controlled trials outside of COVID that stratify patients based on inflammatory subphenotype to guide immunomodulation. It suggests that patients from both subphenotypes should be randomized to avoid missing potential treatment effects in subgroups that may not be expected to benefit the most based on pathophysiological reasoning.

## ACKNOWLEDGMENTS

The Amsterdam UMC COVID-19 Biobank Study Group members are as follows: Michiel van Agtmael (Department of Infectious Diseases), Anne Geke Algera (Department of Intensive Care), Brent Appelman (Department of Infectious Diseases), Floor van Baarle (Department of Intensive Care), Diederik van de Beek (Department of Neurology), Martijn Beudel (Department of Neurology), Harm Jan Bogaard (Department of Pulmonology), Lieuwe Bos (Department of Intensive Care), Michela Botta (Department of Intensive Care), Justin de Brabander (Department of Infectious Diseases), Godelieve Bree (Department of Infectious Diseases), Matthijs C. Brouwer (Department of Neurology), Sanne de Bruin (Department of Intensive Care), Marianna Bugiani (Department of Pathology), Esther Bulle (Department of Intensive Care), Osoul Chouchane (Department of Infectious Diseases), Alex Cloherty (Experimental Immunology), Buis T. P. Buis (Department of Infectious Diseases), Maurits C. F. J. de Rotte (Department of Clinical Chemistry), Mirjam Dijkstra (Department of Clinical Chemistry), Dave A. Dongelmans (Department of Intensive Care), Romein W. G. Dujardin (Department of Intensive Care), Paul Elbers (Department of Intensive Care), Lucas Fleuren (Department of Intensive Care), Suzanne Geerlings (Department of Infectious Diseases), Theo Geijtenbeek (Department of Experimental Immunology), Armand Girbes (Department of Intensive Care), Bram Goorhuis (Department of Infectious Diseases), Martin P. Grobusch (Department of Infectious Diseases), Laura Hagens (Department of Intensive Care), Jorg Hamann (Amsterdam UMC Biobank Core Facility), Vanessa Harris (Department of Infectious Diseases), Robert Hemke (Department of Radiology), Sabine M. Hermans (Department of Infectious Diseases), Leo Heunks (Department of Intensive Care), Markus Hollmann (Department of Anesthesiology), Janneke Horn (Department of Intensive Care), Joppe W. Hovius (Department of Infectious Diseases), Menno D. de Jong (Department of Medical Microbiology), Rutger Koning (Department of Neurology), Endry H. T. Lim (Department of Intensive Care), Niels van Mourik (Department of Intensive Care), Jeannine Nellen (Department of Infectious Diseases), Esther J. Nossent (Department of Pulmonology), Frederique Paulus (Department of Intensive Care), Edgar Peters (Department of Infectious Diseases), Dan A. I. Piña-Fuentes (Department of Neurology), Tom van der Poll (Department of Infectious Diseases), Bennedikt Preckel (Department of Anesthesiology), Jorinde Raasveld (Department of Intensive Care), Tom Reijnders (Department of Infectious Diseases), Michiel Schinkel (Department of Infectious Diseases), Femke A. P. Schrauwen (Department of Clinical Chemistry), Marcus J. Schultz (Department of Intensive Care), Alex Schuurman (Department of Internal Medicine), Jaap Schuurmans (Department of Intensive Care), Kim Sigaloff (Department of Infectious Diseases), Marleen A. Slim (Department of Intensive Care and Infectious Diseases), Patrick Smeele (Department of Pulmonology), Marry Smit (Department of Intensive Care), Cornelis S. Stijnis (Department of Infectious Diseases), Willemke Stilma (Department of Intensive Care), Charlotte Teunissen (Neurochemical Laboratory), Patrick Thoral (Department of Intensive Care), Anissa M. Tsonas (Department of Intensive Care), Pieter R. Tuinman (Department of Intensive Care), Marc van der Valk (Department of Infectious Diseases), Denise P. Veelo (Department of Anesthesiology), Alexander P. J. Vlaar (Department of Intensive Care), Carolien Volleman (Department of Intensive Care), Heder de Vries (Department of Intensive Care), Lonneke A. Vught (Department of Intensive Care and Infectious Diseases), Michèle van Vugt (Department of Infectious Diseases), W. Joost Wiersinga (Department of Infectious Diseases), Dorien Wouters (Department of Clinical Chemistry), and A. H. (Koos) Zwinderman (Department of Clinical Epidemiology, Biostatistics and Bioinformatics).

## APPENDIX A

### Institutional Review Board Approval

Center 1: Amsterdam UMC, Commissie Toetsing Biobanken Amsterdam UMC, Approval number: 2020_065, Approval date: March 06, 2020, Study title: Amsterdam UMC COVID-19 Biobank Study. Center 2: UMC Utrecht, Institutional Review Board (IRB) name: Toetsingscommissie biobanken (TCBio), Approval number: 23-296, Approval date: March 10, 2023, Study title: CARDS-BIO; CARDS-BIO; Secondary Analysis of Molecular Diagnosis and Risk Stratification of Sepsis Biorepository. Center 3: Erasmus MC, IRB name: Niet WMO Toetsingscommissie Erasmus MC, Approval number: 2017-0417 Amendment A-0001, Approval date: November 02, 2025, Study title: Collaboration on Identification, Understanding and Improved Management of Patients With Infectious Diseases (CIUM Study).
